# Active Edible Films Fortified with Natural Extracts: Case Study with Fresh-Cut Apple Pieces

**DOI:** 10.3390/membranes11090684

**Published:** 2021-09-03

**Authors:** Simona Jancikova, Dani Dordevic, Karolina Tesikova, Bojan Antonic, Bohuslava Tremlova

**Affiliations:** Department of Plant Origin Food Sciences, Faculty of Veterinary Hygiene and Ecology, University of Veterinary Sciences Brno, Palackeho tr. 1946/1, 612 42 Brno, Czech Republic; jancikovas@vfu.cz (S.J.); tesikovak@vfu.cz (K.T.); H20364@vfu.cz (B.A.); tremlovab@vfu.cz (B.T.)

**Keywords:** fruit packaging, Golden Delicious, Gala, antioxidant properties, intelligent properties, active properties

## Abstract

The main aim of the study was to prepare the edible films based on carrageenan/chitosan and incorporate them into the following matrices: the natural extracts of *Clitoria ternatea*, *Brassica oleracea*, and *Ipomea batatas*. The films were characterized by TPC (total polyphenols content), antioxidant activity, and textural properties. Experimentally produced films were added in the packaging of freshly cut apple pieces, and the apple pieces were dipped into the films produced from carrageenan and chitosan. The appearance of the samples was monitored, as were antioxidant activity and total polyphenol content. The intelligent properties of films were evaluated too. The polymer type used for the preparation had the highest impact on the prepared films, and CH_LCZ_ (red cabbage extract—*Brassica oleracea*) featured the best antioxidant activity. The intelligent properties were slightly confirmed in samples with the addition of red cabbage. The main finding was that the coating of fresh-cut apples emphasized the possibility to use a carrageenan matrix with the addition of extracts. The samples immersed in this coating type showed higher antioxidant activity as well as a superior color when compared to that of chitosan coated apple samples.

## 1. Introduction

Edible packaging can be defined as matrices based on proteins, lipids, and polysaccharides [1]. They have a long history—for example, the preparation of wax coating on citrus fruits, and recently, scientists tried to find innovative edible packaging based on new types of polysaccharides or by incorporating byproducts, natural extracts, oils, nanoparticles, etc. [2,3].

Commonly used polysaccharide materials for a film or coating preparation are carrageenan and chitosan. Carrageenan has three different chemical forms—ι, κ, and λ; extracted from red seaweeds. The κ-carrageenan-based edible films are stronger than the edible films based on ι-carrageenan [4,5]. κ-carrageenan consists of 3-linked β-D-galactopyranose 4-sulphate and 4-linked 3,6-anhydro-α-D-galactopyranose [6]. On the other hand, the chitosan is prepared from chitin extracted from the shells of crabs and shrimps and is characterized by antimicrobial properties and biodegradability [7,8,9]. The structure of chitosan is very similar to cellulose—there are units of β-(1-4) linked D-glucose: more specifically, β-(1,4) linked 2-amino-2-deoxy-β-D-glucopyranose [10].

As mentioned, the natural extracts and other compounds can be added to edible films and the films can gain the active properties, thereby improving the shelf life of food by the migration of bioactive compounds or intelligent properties. This also allows for detecting the condition of food by changing the color of the packaging [11]. The blue tea also known as butterfly pea or Asian pigeowings (*Clitoria ternatea*) is known for its intensely blue color, which is caused by the high anthocyanin content, and these pigments are sensitive to the environmental pH [12,13]. The butterfly tea extract is often used as a natural food colorant and in the pharmaceutical and cosmetics industries [14,15]. The color of anthocyanins in *Clitoria ternatea* flowers is highly dependent on pH [16]. The extract from red cabbage can be used as a dietary supplement, and it is often used as a source of natural dyes for food products [17]. The big advantage of red cabbage extract is that the red cabbage’s anthocyanins can be coloured in a very broad pH range compared to that of anthocyanins from grape skin or elderberry [18]. Parts of the sweet potato (*Ipomea batatas*), such as the roots, stems, and leaves, are a good source for various compounds: vitamins, essential amino acids, natural antioxidants. However, the amount of bioactive compounds is dependent on their cultivar color and environmental and processing conditions [19]. Huang et al. [20] found that the phytochemicals present in sweet potatoes have a significant effect on antioxidant and anticancer activities. 

The specific examples of the main compounds occurring in natural extracts include phenolic compounds, such as gallic acid and caffeic acid, vitamin C, and carotenoids [21]. Anthocyanins, along with chalcones, flavones, and flavonols, belong to the flavonoids, whose basic structure is C6-C3-C6 [22].

Edible packaging is used for the packaging of fresh-cut fruits and vegetables in the form of edible coating. The sample is immersed in the film-forming solution, and it creates a protective coating directly on the surface of food [23]. Previous studies indicated that fresh-cut apple pieces can be packaged in different types of packaging with the addition of essential oils or nanoparticles [24,25]. Similarly, other types of fruits can be packed or coated with chitosan and carrageenan matrices with the addition of natural extracts or essential oils [26,27].

In our study, the edible films based on κ-carrageenan and chitosan were prepared, and the natural extracts from red cabbage (CZ), sweet potato (BT), and blue tea (MC) were added. The characterization of the prepared films was evaluated along with the application of the edible films as intelligent packaging and the active packaging during storage of fresh-cut apple pieces.

## 2. Materials and Methods

### 2.1. Materials

The chemicals for the analyses were purchased from Sigma–Aldrich (Munich, Germany) and PENTA chemicals (Prague, Czech Republic). Fruits and plants used for sample preparations are described below.

### 2.2. Preparation of Natural Extracts

The natural extracts were prepared from red cabbage (CZ) (Brassica oleracea var. capitata f. Rubra), sweet potato (BT) (Ipomoea batatas), and blue tea (MC) (Clitoria ternatea). The red cabbage and sweet potato were purchased at local market Billa (Brno, Czech Republic), and the blue tea was purchased at MANUtea (Ludgerovice, Czech Republic). Extracts from red cabbage and sweet potato were prepared as follows: 10 g of cut samples were weighted, and 100 mL of hot distilled water was added; after 10 min of extraction, the extracts were filtered and used for the preparation of films and film-forming solutions.

### 2.3. Preparation of Edible Packaging (Film-Forming Solution)

The recipes used to produce chitosan films are described in Table 1. The preparation: 0.5 g of low molecular chitosan (CHL) and 45 mL of 1% lactic acid were added, or else 0.5 g of κ-carrageenan (KAR) was weighted and 45 mL of distilled water was added. The mixture was heated and then shaken on a magnetic stirrer for 15 min (50 °C, 500 rpm). Next, 0.25 g of glycerol was added, followed by 5 min of stirring. In the case of films with the addition of extracts, 1% lactic acid was replaced by the extracts in 20% concentration. The film-forming solutions were used for the coating of fresh-cut apples, and they were placed in Petri dishes and dried for 24 h (film preparation technique).

### 2.4. Thickness

The thickness of prepared edible packaging was analyzed by micrometer Mitotuyo M310-25 (Kawasaki, Japan). The calculation of average thickness was completed after measuring in five different places.

### 2.5. Textural Properties

The textural properties—breaking strain (%) and strength (MPa)—were analyzed by texturometer TA.XT plus (Godalming, UK). The ASTM International test method, ASTM D882-02, was used for measuring. The prepared films were cut into dimensions of 5 cm × 1 cm, and each sample was analyzed four times.

### 2.6. Preparation of Apple Samples

Two kinds of apples were used: green apples, Malus domestica ’Golden Delicious’, harvested in Italy, and red apples, Malus domestica ‘Gala’, harvested in the Czech Republic. Both kinds of apples were purchased at the local Tesco market (Brno, Czech Republic). The apples were peeled and cut into a dimension of 1 cm × 1 cm × 1 cm, and then immediately used for another analysis.

### 2.7. Determination of Intelligent Properties

To determine intelligent properties, cut apples were put into a plastic bag, and a piece of chitosan film (1 cm × 1 cm) was placed directly in the bag and another was placed outside on the surface as the control sample. The samples were stored in a refrigerator at 4 °C for 11 days. The pH and browning index served as the detection factor for the films’ intelligent properties.

### 2.8. Application of Packaging on Fresh Cut Apple Pieces

Fresh-cut apples were immersed in film-forming solutions for 1 min, and then the samples were moved to plastic boxes. After a short drying period, they were then put in the refrigerator at 4 °C for 7 days. The samples were subsequently analyzed using the following methods: FRAP (Ferric Reducing Antioxidant Power), DPPH (2,2,-diphenyl-1-picrylhydrazyl), total polyphenols content, and browning index.

### 2.9. pH Determination

The pH of apple samples was evaluated in homogenized samples by pH meter (GRYF 259, Havlickuv Brod, Czech Republic) in triplicates.

### 2.10. Browning Index

The determination of browning index was performed according to Nollet [28], with slight modifications. Two grams of apple pieces were weighted and 5 mL of distilled water was added. The samples were centrifuged and filtered, and the absorbance was measured at 420 nm on the spectrophotometer (CE7210 DIET-QUEST, Cambridge, UK).

### 2.11. FRAP (Ferric Reducing Antioxidant Power)

Antioxidant activity was determined by FRAP method according to Behbahani et al. [29], with slight modifications. The 0.1 g of homogenized sample was put into a flask and 20 mL of 75% methanol was added. The sample was placed in an ultrasound water bath and extracted for 30 min. After that, the samples were filtered and mixed with a working solution (300 mM acetic buffer, 10 mM TPTZ, and 20 mM FeCl_3_·3H_2_O). The samples were incubated in the dark for 8 min, and then the absorbance was measured at 593 nm on spectrophotometer (CE7210 DIET-QUEST, Cambridge, UK). The results were expressed as µmol Trolox/g of sample.

### 2.12. DPPH (2,2-diphenyl-1-picrylhydrazyl)

The determination of DPPH was done according to Adilah et al. [30], with slight modifications. The 0.1 g of homogenized sample was weighted in a flask, and 20 mL of ethanol was added. After 30 min of incubation in ultrasound water bath, the samples were filtered, and 3 mL were mixed with 1 mL of 0.1 mM DPPH solution in ethanol. The procedure was followed by 30 min of incubation in the dark, and then the absorbance was measured at 517 nm on the spectrophotometer (CE7210 DIET-QUEST, Cambridge, UK). The results were expressed as DPPH scavenging activity:

DPPHscavenging activity (%) = [(AbsDPPH − Abssample/AbsDPPH)] × 100

### 2.13. Total Polyphenols Content

Total polyphenol content was determined via Folin–Ciocalteau solution according to Tomadoni et al. [31], with slight modifications. One gram of homogenized sample was weighted in the beaker, and 10 mL of distilled water was added. After 10 min on laboratory shaker, the samples were filtered, and 1 mL of sample was added to a volumetric flask, followed by the addition of Folin–Ciocalteau (1:10) and 7.5% Na_2_CO_3_, and then the mixture was incubated in the dark for 30 min. The absorbance was measured at 765 nm on the spectrophotometer (CE7210 DIET-QUEST, Cambridge, UK), and the results were expressed as mg gallic acid/g.

### 2.14. Statistical Analysis

IBM SPSS program was used to perform the statistical analysis. The one-way ANOVA test was used for the determination of statistically significant differences at *p* < 0.05. They were further analyzed using the parametric Tukey’s post hoc test (when the Levene’s test showed equal variances *p* > 0.05) and nonparametric Games–Howell post hoc test (when Levene’s test showed unequal *p* < 0.05). For the determination of statistical differences between two measurements, the *t*-test was used.

## 3. Results and Discussion

### 3.1. The Characterization of Prepared Packaging

The total polyphenols content and antioxidant properties of prepared packaging are summarized in Table 2. Lower values of TPC, same as FRAP and DPPH, were found in carrageenan samples, and the CHL and KAR samples, with the addition of same extract, were in all cases statistically significantly different (*p* < 0.05).

TPC increased with the addition of extracts, and the statistically significant differences (*p* < 0.05) were found between all samples. TPC was highest in the sample CH_LCZ_ (0.125 ± 0.001 mg gallic acid/g). The values of FRAP method were the highest in samples CH_LMC_, CH_LBT,_ and CH_LCZ_; these samples were statistically different (*p* < 0.05) in comparison with that of the CH_L_ and KAR samples.

DPPH method corresponded with TPC; the highest DPPH scavenging activity was in the sample CH_LCZ_, which was also statistically different (*p* < 0.05) from other samples. The differences between FRAP and DPPH method are based on the principle of these methods, as the FRAP method uses chemicals with low pH value (3.6). The DPPH method is based on radical principle, and the FRAP method indicates the new formed ferrous ions [32]. The other reason why the antioxidant methods do not correspond with one another is that different kinds of antioxidants can be found in the examined samples, reacting differently based on the method used [33]. The differences between increasing antioxidant activity based on what matrix is used (KAR or CH_L_) can be explained by the antioxidant activity of carrageenan, which is supported by the presence and number of sulfated groups [4,34]. On the other hand, the antioxidant activity of chitosan is caused by the presence of nitrogen at the C2 position of chitosan, and it can scavenge various free radicals [35].

Prepared packaging was analyzed by the methods for determination of physical properties (thickness, breaking strain, and strength), and results are shown in Table 3.

The results indicate that the addition of extracts in both basic matrices based on carrageenan and chitosan increases the thickness of produced films; however, when comparing all carrageenan samples, only the thickness of KAR_BT_ was statistically significantly (*p* < 0.05) higher than in that of KAR (the control sample without extract). No statistically significant (*p* > 0.05) difference was found between chitosan samples and CH_L_ (the control sample without extract).

The breaking strain of films were lower in samples prepared with carrageenan than with chitosan, but statistically significant differences were not observed. The carrageenan samples, after the addition of extracts, had higher values of breaking strain than KAR, but not KAR_BT_. In the case of chitosan, the addition of CZ, MC, and BT decreased the breaking strain value.

The strength of films corresponded with the breaking strain. The carrageenan samples were stronger than chitosan samples, regardless of the extract added to the sample, and these results were also statistically significant (*p* < 0.05) different, except for samples with the addition of blue tea. The strength was lower in KAR_MC_, KAR_CZ,_ and KAR_BT_ than in that of the KAR sample, but the difference was not statistically significant (*p* > 0.05). The same conclusion can be observed when comparing CH_L_ and chitosan-based samples with the addition of extracts. The strength of prepared samples was not affected by the type of natural extract, but only by the used basic matrix/polymer. The explanation of different textural properties can be affected by the different pH since the preparation of chitosan films included 1% lactic acid [36,37].

### 3.2. Intelligent Properties of Chitosan Films during Storage of Apples

**Figure 1:**
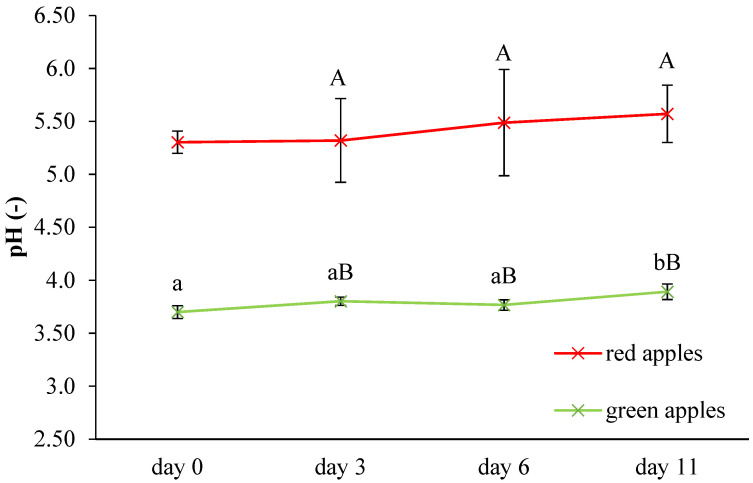
Determination of red and green apples’ pH during storage (uppercase letters indicate statistically significant differences (*p* < 0.05) between red and green apples; lowercase letters indicate significant differences (*p* < 0.05) between days of storage).

The results of pH monitoring during storage of fresh-cut apple pieces are summarized in Figure 1. The pH of red apples was statistically significantly (*p* < 0.05) higher than pH of green apples; this finding was observed across the entire storage time. 

pH of green, fresh-cut apple pieces mostly increased (from 3.70 to 3.89), and after 11 days, a statistically significant (*p* < 0.05) difference was observed. In both apple samples, pH increased during storage, but in the case of red fresh-cut apple pieces, there were no statistically significant differences.

The increasing pH value during storage of Golden Delicious fresh-cut apples caused by metabolic processes were confirmed by previous works [38,39]. Usually, red apples have higher pH values than that of green apples, and these parameters are very important for consumers since they appreciate the balance of sweetness and acidity [40]. pH of apples is mainly affected by the storage of malic acid in vacuoles, and a high amount of this organic acid can change an apple’s pH [41,42]. Piagentini and Pirovani [43] found that red apples have a significantly (*p* < 0.05) lower amount of malic acid than green apples.

**Figure 2:**
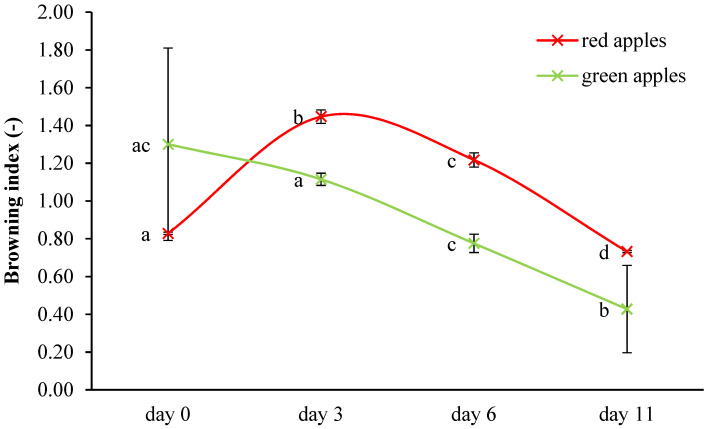
Determination of apples’ browning index (uppercase letters indicate statistically significant differences (*p* < 0.05) between red and green apples; lowercase letters indicate significant differences (*p* < 0.05) between days of storage).

Figure 2 compares the results obtained by the determination of browning index. The browning index is expressed as the absorbance at 420 nm; in the samples of fresh cut red apples, the results did not show a certain trend, but until day 3, the browning index increased, and after this, the results decreased until day 11. Statistically significant (*p* < 0.05) differences were found between all storage days of red apples. The different trend was found in samples of fresh-cut green apples; the browning index decreased during storage time, but notably, from day 0, there was no statistically significant (*p* < 0.05) different result until day 11. During the storage of apples, the following trends were observed by other authors: in the first 180 min, the browning index increased, but after 24 h, it decreased [44]. Remorini et al. [45] determined that after 48 h of storage, the browning index increased and then decreased. The browning of apples can be enzymatic or nonenzymatic. Nonenzymatic processes include caramelization, ascorbic acid degradation, and Maillard reaction [46]; enzymatic browning is mainly caused by the polyphenol oxidases [47].

**Figure 3:**
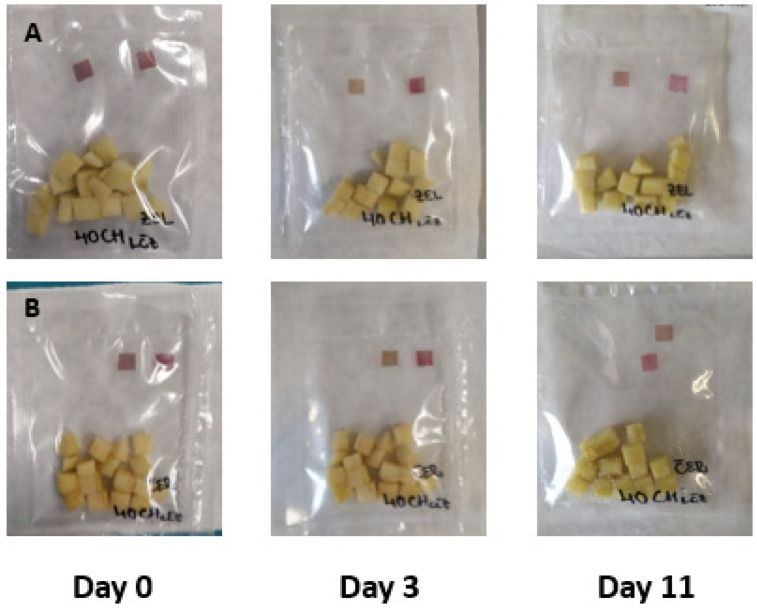
Green (**A**) and red (**B**) apples packaged with pieces of film made with the addition of red cabbage extract.

**Figure 4:**
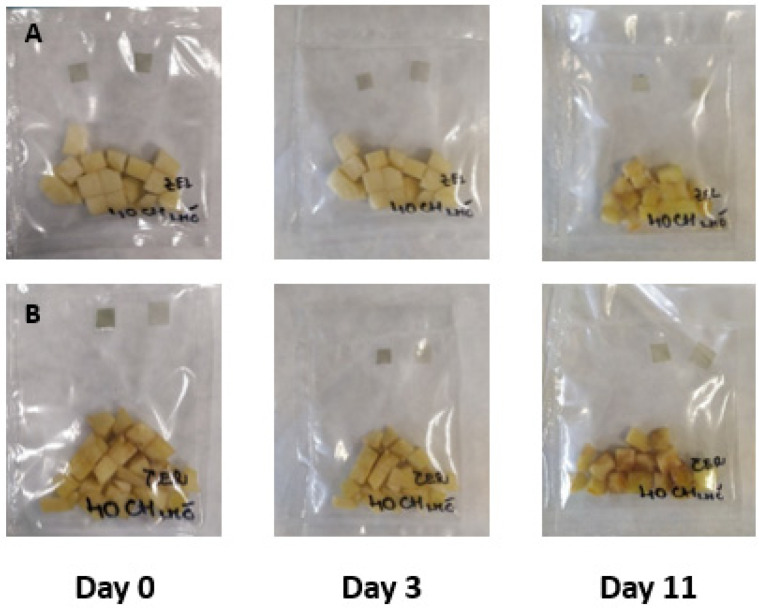
Green (**A**) and red (**B**) apples packaged with pieces of film made with the addition of blue tea.

**Figure 5:**
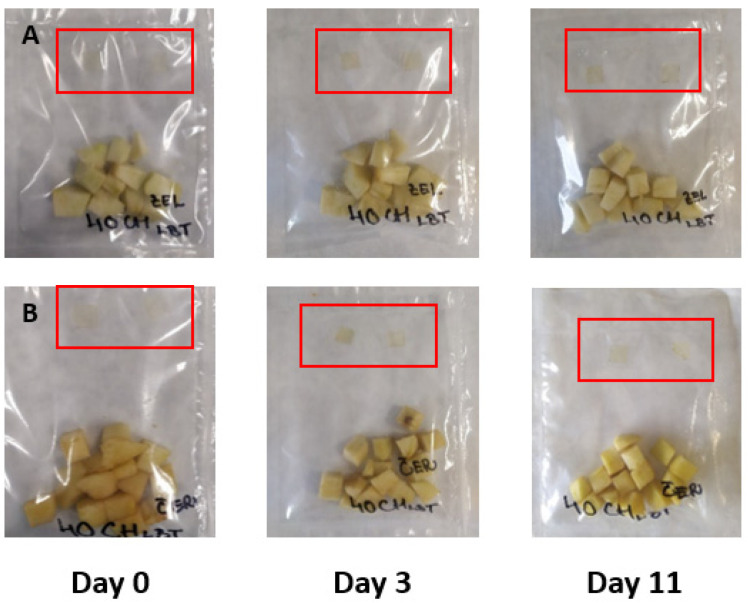
Green (**A**) and red (**B**) apples packaged with pieces of film made with the addition of sweet potato extract.

**Figure 6:**
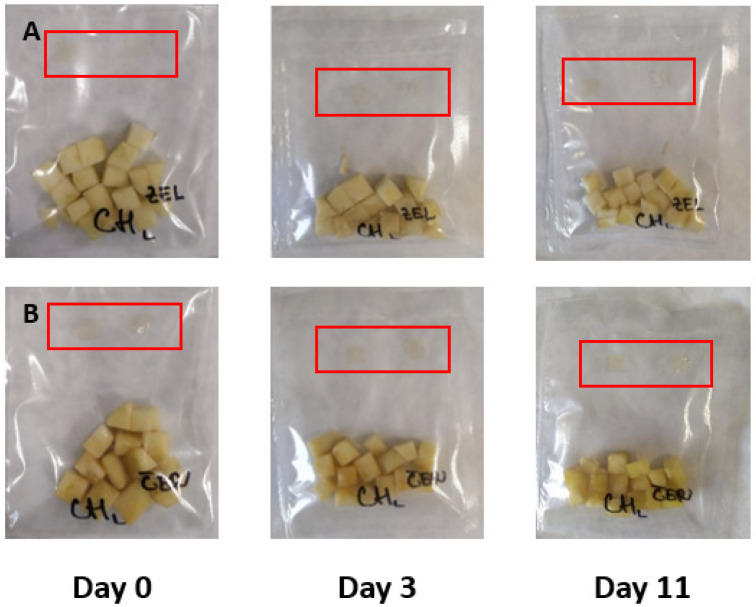
Green (**A**) and red (**B**) apples packaged with pieces of film made without the addition of extracts.

The results of the experiment based on the addition of small edible films pieces prepared in sealed bags with fresh-cut apple pieces are summarized in Figure 3, Figure 4, Figure 5 and Figure 6. The film pieces on the left-side are outside of the sealed bags, and the film pieces on the right-side are inside of the sealed bags. 

Figure 3 shows the color changes of chitosan films with the addition of red cabbage extract during 11 days of storage. The color changes in day 0 were invisible, but the film pieces put inside a sealed bag changed the color towards a darker shade of purple after the beginning of the experiment. Several reports showed that red cabbage is a good source of anthocyanins and can change color in different pH environment. Anthocyanins in red cabbage have red color in acidic environment, and at pH 6, the color changes to purple, and from blue to yellow-green color in a basic environment [9]. The anthocyanins’ color change is based on pH because in pH under 3, the chemical structure is flavylium cation (red color); at mildly acidic pH, it is carbinol pseudobase (without color); at neutral pH, quinonoidal base (purple), and at alkaline pH, chalcone (yellow) [48,49].

As shown in Figure 4 (color changes of chitosan films with the addition of blue tea extracts were monitored), the changes were not visible. Also during storage, both of the chitosan pieces (inside, same as outside) lost color intensity. The blue tea is a good source of anthocyanins pigment, and blue tea extracts can change color with different pH values [12].

Figure 5 shows the results of chitosan films’ color changes with the addition of sweet potato extract. The changes during storage were not visible, but the color is slightly different in comparison with that of the control samples.

Figure 6 summarized the results of intelligent properties of control chitosan films. The changes, same as the film itself, are invisible due to the absence of natural extracts.

To summarize the results examining intelligent properties of experimentally produced films with the addition of red cabbage, blue tea, and sweet potato extracts, changes in pH values during storage of fresh-cut Golden Delicious apple pieces were not so high; the color of films was not exclusively visible, meaning that consumers would not notice unambiguous color differences. However, the addition of natural extracts to chitosan films affected the color, as shown when comparing control films (Figure 6) with the films showed in Figure 3, Figure 4 and Figure 5.

### 3.3. Packaging of Apples by Enriched Chitosan and Carrageenan Coatings

**Figure 7:**
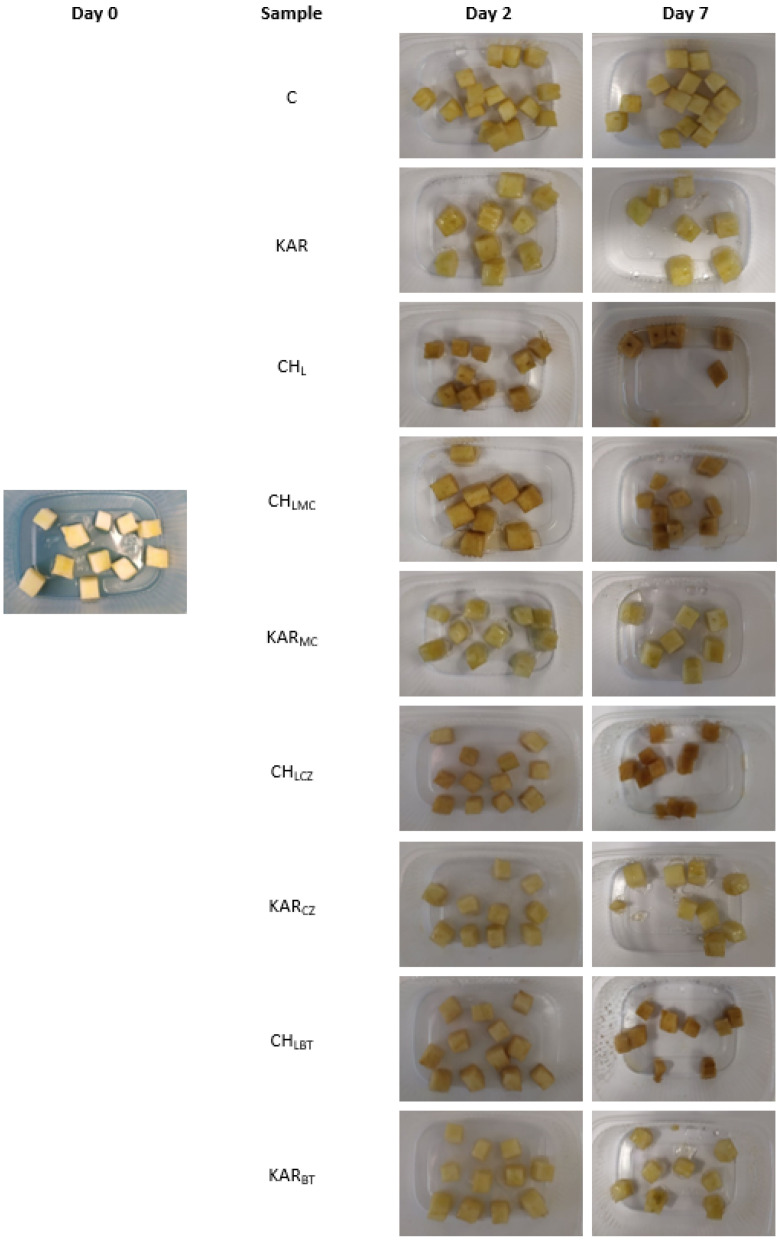
Appearance of packaged fresh-cut Golden Delicious apple pieces.

The results of appearance of red apples immersed in film-forming solution with the addition of natural extracts are shown in Figure 7. Importantly, the fresh-cut Golden Delicious apples immersed in chitosan-based film-forming solutions had high brownish color after 2 days, and the brown color continuously became darker up to day 7. Chitosan-based films, when compared to that of control samples after 7 days of storage, were less brown. The samples packaged in carrageenan film-forming solution had a similar appearance on days 2 and 7, and color changes were not observed.

Antioxidant activity of fresh-cut apple pieces packed in experimentally produced edible films (in refrigerator for 7 days) is shown in Table 4 and Table 5. Fresh-cut green apple samples had the highest antioxidant activity determined by FRAP method; after 2 days of storage with the samples immersed in KAR film, the result was statistically significant (*p* < 0.05) different from other analyzed samples. The second highest results were observed in samples C, CH_LMC,_ and KAR_MC_ on day 2. Anthocyanins are present in plants, and they act as antioxidants, same as pigments; red cabbage is a good source of anthocyanins [9,50]. The samples packaged in films with the addition of red cabbage and blue tea extract had higher antioxidant activity and anthocyanin content [12].

Previous research found that not all fresh-cut apple piece samples had a higher antioxidant capacity when packed in alginate and pectin coating with essential oils (eugenol, citral) than in that of control samples without packaging [24]. Antioxidant activity of carrageenan packaging was lower than in that of chitosan-based packaging, but fresh-cut apple pieces packed in carrageenan packaging had a higher antioxidant activity. 

Table 6 compares the results of total phenol content of fresh-cut green apple pieces immersed in the prepared film-forming solutions across 7 days of storage. The total phenol content was higher in the fresh-cut apple pieces packaged in carrageenan than in that of chitosan. The highest phenol content after 7 days of storage was found in samples C, KAR, KAR_MČ,_ and KAR_ČZ_. A higher TPC in control samples (without films) can be explained by higher percentage of fruit material. After 2 and 7 days, the amount of TPC in samples packaged in chitosan films (regardless of the addition of extract) decreased, but in the samples packaged in carrageenan films, it increased. Notably, there are no general rules for decreasing or increasing TPC during storage. The increase of TPC is affected by the activity of fenylalaninammoniumlyase, the enzyme supporting the synthase of phenols, and its activity is higher during cutting and peeling. On the other hand, the decrease of TPC is caused by polyphenoloxidase, which causes the browning of apples. These reactions can be affected by the application of edible coating and also by the presence of antioxidants [23]. Cofelice et al. [23] did not observe the trend of TPC increases or decreases during storage of fresh-cut apple pieces in coatings with essential oil.

The results of browning index (fresh-cut apple pieces immersed in prepared film-forming solutions with specific extracts) are shown in Table 7. The results emphasized that after 2 days of storage, the browning index decreased in all samples, but in the last day of storage (day 7), the highest browning index was found in the control sample, and there were also statistically significant (*p* < 0.05) differences between all tested samples. Conversely, previous studies observed the increase of the browning index during storage of fresh-cut apples [25].

## 4. Conclusions

The properties of experimentally prepared edible films showed that the total polyphenol content increased with the addition of extracts, but there were significant differences not only caused by the presence of natural extracts in edible films, but also by the polymer used as the basic matrix—κ-carrageenan or chitosan. Another finding that can be emphasized is that the strength of the κ-carrageenan films was not significantly affected by the addition of extracts. The κ-carrageenan films were stronger than chitosan films. The intelligent properties of chitosan films were most visible with the addition of red cabbage extract; in the case of blue tea and sweet potato extract additions, the differences were not visible. The packaging of fresh-cut apple pieces showed different behavior in the samples; when the apple pieces were coated in chitosan, their color was more brown than in that of κ-carrageenan coating. In the most cases, the carrageenan coating samples showed better antioxidant properties and greater total polyphenol content than that of apple samples immersed in chitosan coating. Certainly, these findings and observations can serve as a good starting point for future experiments and in the modification of edible film production. 

## Figures and Tables

**Table 1:** Composition of prepared edible films.

Sample	Composition
KAR	0.5 g κ-carrageenan + distilled H_2_O + glycerol
CH_L_	0.5 g chitosan + distilled H_2_O + glycerol
CH_LMC_	0.5 g chitosan + distilled H_2_O + 20 % *Clitoria ternatea* extract + glycerol
KAR_MC_	0.5 g κ-carrageenan + distilled H_2_O + 20 % *Clitoria ternatea* extract + glycerol
CH_LCZ_	0.5 g chitosan + distilled H_2_O + 20 % *Brassica oleracea* extract + glycerol
KAR_CZ_	0.5 g κ-carrageenan + distilled H_2_O + 20 % *Brassica oleracea* extract + glycerol
CH_LBT_	0.5 g chitosan + distilled H_2_O + 20 % *Ipomea batatas* extract + glycerol
KAR_BT_	0.5 g κ-carrageenan + distilled H_2_O + 20 % *Ipomea batatas* extract + glycerol

**Table 2:** Total polyphenol content and antioxidant properties (Ferric Reducing Antioxidant Power (FRAP) and DPPH) of edible films.

Sample	TPC (mg Gallic Acid/g)	FRAP (µmol Trolox/g)	DPPH (%)
KAR	0.008 ± 0.001 ^a^	0.52 ± 0.02 ^a^	0.41 ± 0.35 ^a^
CHL	0.020 ± 0.001 ^b^	0.73 ± 0.02 ^b^	2.63 ± 0.07 ^b^
CHL_MC_	0.090 ± 0.001 ^c^	1.46 ± 0.06 ^cd^	3.12 ± 0.12 ^c^
KAR_MC_	0.033 ± 0.000 ^d^	0.84 ± 0.08 ^ab^	1.01 ± 0.11 ^a^
CHL_CZ_	0.125 ± 0.001 ^e^	1.26 ± 0.04 ^c^	6.29 ± 0.07 ^d^
KAR_CZ_	0.014 ± 0.000 ^f^	0.85 ± 0.08 ^ab^	3.03 ± 0.04 ^c^
CHL_BT_	0.111 ± 0.001 ^g^	1.49 ± 0.02 ^d^	2.32 ± 0.13 ^b^
KAR_BT_	0.066 ± 0.000 ^h^	0.60 ± 0.05 ^ab^	0.92 ± 0.24 ^a^

Letters in superscript indicate statistically significant (*p* < 0.05) differences between rows.

**Table 3:** Physical properties of prepared packaging.

Sample	Thickness (mm)	Breaking Strain (%)	Strength (MPa)
KAR	0.09 ± 0.01 ^a^	96.43 ± 2.89	0.13 ± 0.01 ^a^
CHL	0.17 ± 0.05	127.66 ± 10.85	0.03 ± 0.00 ^b^
CHL_MC_	0.19 ± 0.03 ^b^	107.80 ± 8.63	0.09 ± 0.04
KAR_MC_	0.10 ± 0.01 ^ac^	97.77 ± 1.61	0.12 ± 0.00 ^a^
CHL_CZ_	0.17 ± 0.02 ^b^	108.94 ± 9.05	0.04 ± 0.01 ^bc^
KAR_CZ_	0.10 ± 0.01 ^ac^	102.30 ± 3.32	0.12 ± 0.01 ^a^
CHL_BT_	0.20 ± 0.04 ^b^	96.85 ± 3.11	0.06 ± 0.00 ^c^
KAR_BT_	0.11 ± 0.00 ^c^	93.56 ± 2.44	0.11 ± 0.01 ^a^

Letters in superscript indicate statistically significant (*p* < 0.05) differences between rows.

**Table 4:** FRAP antioxidant activity (expressed as µmol Trolox/g) of Golden Delicious apples packaged in prepared film-forming solutions.

Day 0	Day 2		Day 7	
3.07 ± 0.02	C	2.06 ± 0.03 ^a^	C	11.02 ± 0.07 ^a^
	KAR	2.80 ± 0.05 ^b^	KAR	0.18 ± 0.02 ^b^
	CH_L_	0.40 ± 0.08 ^cf^	CH_L_	0.00 ± 0.00 ^cd^
	CH_LMC_	1.76 ± 0.09 ^ad^	CH_LMC_	0.17 ± 0.03 ^bcd^
	KAR_MC_	1.42 ± 0.09 ^d^	KAR_MC_	0.00 ± 0.00 ^c^
1.33 ± 0.04	CH_LCZ_	0.25 ± 0.02 ^c^	CH_LCZ_	0.00 ± 0.00 ^c^
	KAR_CZ_	0.93 ± 0.03 ^e^	KAR_CZ_	2.55 ± 0.01 ^e^
	CH_LBT_	0.00 ± 0.00 ^f^	CH_LBT_	0.40 ± 0.04 ^f^
	KAR_BT_	0.00 ± 0.00 ^f^	KAR_BT_	0.89 ± 0.01 ^g^

Letters in superscript indicate statistically significant (*p* < 0.05) differences between rows.

**Table 5:** DPPH scavenging activity (%) results of Golden Delicious apples packaged in prepared film-forming solutions.

Day 0	Day 2		Day 7	
20.63 ± 0.32	C	33.16 ± 1.39 ^a^	C	35.58 ± 0.16 ^a^
	KAR	17.70 ± 0.06 ^b^	KAR	24.50 ± 0.12 ^b^
	CH_L_	4.77 ± 1.26 ^hc^	CH_L_	1.87 ± 0.18 ^c^
	CH_LMC_	18.07 ± 1.21 ^be^	CH_LMC_	2.18 ± 0.28 ^c^
	KAR_MC_	10.79 ± 1.07 ^dfgh^	KAR_MC_	22.72 ± 0.14 ^d^
7.78 ± 0.15	CH_LCZ_	14.98 ± 0.27 ^ef^	CH_LCZ_	2.27 ± 0.22 ^c^
	KAR_CZ_	18.15 ± 0.14 ^b^	KAR_CZ_	25.86 ± 0.14 ^e^
	CH_LBT_	9.85 ± 0.18 ^cg^	CH_LBT_	4.86 ± 0.17 ^f^
	KAR_BT_	4.99 ± 0.03 ^h^	KAR_BT_	11.97 ± 0.19 ^g^

Letters in superscript indicate statistically significant (*p* < 0.05) differences between rows.

**Table 6:** TPC (expressed as mg gallic acid/g) of Golden Delicious apples packaged in prepared film forming solutions.

Day 0	Day 2		Day 7	
0.312 ± 0.000	C	0.330 ± 0.000 ^a^	C	0.477 ± 0.002 ^a^
	KAR	0.176 ± 0.001 ^b^	KAR	0.351 ± 0.001 ^b^
	CH_L_	0.097 ± 0.001 ^c^	CH_L_	0.075 ± 0.000 ^c^
	CH_LMC_	0.135 ± 0.000 ^d^	CH_LMC_	0.079 ± 0.000 ^d^
	KAR_MC_	0.168 ± 0.000 ^e^	KAR_MC_	0.201 ± 0.000 ^e^
0.379 ± 0.000	CH_LCZ_	0.128 ± 0.000 ^f^	CH_LCZ_	0.071 ± 0.001 ^c^
	KAR_CZ_	0.218 ± 0.000 ^g^	KAR_CZ_	0.287 ± 0.004 ^f^
	CH_LBT_	0.183 ± 0.000 ^h^	CH_LBT_	0.122 ± 0.002 ^g^
	KAR_BT_	0.227 ± 0.001 ^i^	KAR_BT_	0.193 ± 0.000 ^h^

Letters in superscript indicate statistically significant (*p* < 0.05) differences between rows.

**Table 7:** Browning index (-) of Golden Delicious apples packaged in prepared film forming solutions.

Day 0	Day 2		Day 7	
1.58 ± 0.02	C	0.43 ± 0.00 ^a^	C	2.13 ± 0.10 ^a^
	KAR	0.94 ± 0.01 ^b^	KAR	1.04 ± 0.01 ^b^
	CH_L_	0.37 ± 0.00 ^c^	CH_L_	0.71 ± 0.00 ^c^
	CH_LMC_	1.16 ± 0.01 ^d^	CH_LMC_	1.38 ± 0.00 ^d^
	KAR_MC_	0.60 ± 0.00 ^e^	KAR_MC_	0.54 ± 0.00 ^e^
1.32 ± 0.01	CH_LCZ_	0.60 ± 0.01 ^d^	CH_LCZ_	0.59 ± 0.00 ^f^
	KAR_CZ_	1.24 ± 0.00 ^f^	KAR_CZ_	1.34 ± 0.01 ^g^
	CH_LBT_	0.95 ± 0.01 ^b^	CH_LBT_	0.37 ± 0.00 ^h^
	KAR_BT_	1.22 ± 0.00 ^g^	KAR_BT_	1.00 ± 0.00 ^i^

Letters in superscript indicate statistically significant (*p* < 0.05) differences between rows.

## Data Availability

Not applicable.
